# Rapidly Exacerbating Autoimmune Hemolytic Anemia Together With Marked Cytokine Storm Triggered by Pneumonia Infection: A Case Report

**DOI:** 10.3389/fimmu.2020.574540

**Published:** 2020-09-15

**Authors:** Yuichiro Iwamoto, Takatoshi Anno, Katsumasa Koyama, Ryo Shirai, Hideaki Kaneto, Koichi Tomoda

**Affiliations:** ^1^Department of General Internal Medicine 1, Kawasaki Medical School, Okayama, Japan; ^2^Department of Diabetes, Endocrinology and Metabolism, Kawasaki Medical School, Kurashiki, Japan

**Keywords:** pneumonia, autoimmune hemolytic anemia, hypercytokinemia, cytokine storm, Th2-dominant conditions

## Abstract

**Background:** Autoimmune hemolytic anemia (AIHA) is caused by hemolysis induced by the reaction of autoantibodies with red blood cells. AIHA is usually classified as either warm antibody or cold antibody-mediated AIHA. In addition, AIHA caused by infection is classified as secondary AIHA. It is well-known that alteration of various cytokine levels is closely associated with a variety of disorders such as infectious diseases. In addition, it is known that IL-10/ IL-12 imbalance is an indicator of Th2-dominat conditions and a progressive marker of AIHA.

**Case presentation:** A 82-year-old Japanese man was brought to the emergency room with pneumonia and heart failure. After admission, we started antibiotics therapy. Next day, he had symptoms of jaundice and his total bilirubin level was elevated. Five days after admission, his inflammation markers were further elevated and he had hemolytic anemia. We finally diagnosed him as severe warm-type AIHA associated with pneumoniae infection. Seven days after admission, his severe leucocytosis was further aggravated, and then he suddenly had cardiac arrest and respiratory failure, and finally died of multiple organ failure. Unfortunately, we failed to rescue him from severe AIHA induced by pneumonia infection.

**Conclusions:** Herein, we report a subject with pneumonia-triggered warm-type AIHA together with marked cytokine storm. IL-18 levels were markedly elevated without elevation of IL-12 levels. In addition, IL-10/IL-12 imbalance was observed. These data suggest that once marked cytokine storm is induced, infection-induced AIHA is exacerbated very rapidly.

## Background

Autoimmune hemolytic anemia (AIHA) is caused by hemolysis induced by the reaction of autoantibodies with red blood cells (RBC) (1). Anti-erythrocyte antibodies cause RBC to burst as hemolytic anemia, although anti-erythrocyte antibodies are generated by the patient's own immune system. Therefore, AIHA is a severe and sometimes fatal disease. AIHA is usually classified as either warm antibody or cold antibody-mediated AIHA (2). The diagnosis is usually based on the presence of hemolytic anemia and serological evidence of anti-erythrocyte antibodies, detectable with the direct antiglobulin test (DAT) and cold agglutination (3). In general, subjects with warm-type AIHA show positive reaction in DAT with anti-IgG antisera and/or anti-C3d, and those with cold-type AIHA show positive reaction in cold agglutination. The exact incidence of AIHA in adults is unclear. One of the causes for AIHA is serious infections such as mycoplasma pneumoniae and viruses (4, 5). This type of AIHA occurs when normal RBC get destroyed by the infection or by anti-erythrocyte antibodies.

It is well-known that alteration of various cytokine levels is closely associated with a variety of disorders such as infectious diseases. Among various cytokines, interleukin (IL)-18 is a unique cytokine; IL-18 functions as Th1 cytokine in the presence of IL-12 but functions as Th2 cytokine in the absence of IL-12 (6). Th1 cells are involved in “cell-mediated” immunity, which usually deals with infections by viruses and certain bacteria. Th2 cells are involved in “humoral-mediated” immunity, which deals with bacteria, toxins and allergens. In addition, it is known that IL-10/ IL-12 imbalance is an indicator of Th2-dominat conditions and a progressive marker of AIHA (7, 8).

In this report, we show a subject who had pneumonia infection and rapidly exacerbating warm-type AIHA accompanied by marked cytokine storm under Th2-dominant conditions.

## Case Presentation

An 82-year-old Japanese man was brought to the emergency room with symptoms of high fever, dyspnea and general fatigue over 3 days. His vital signs were: temperature, 38.7°C; blood pressure, 104/74 mmHg; heart rate, 82 bpm; oxygen saturation, 94 % (O_2_4 L/min). Inflammation markers were markedly elevated (Table 1): white blood cell (WBC), 9,030/μL (neutrophil, 90.0 %); C-reactive protein (CRP), 10.13 mg/dL; procalcitonin, 0.55 ng/mL. Another laboratory data were as follows: RBC, 441 × 10^4^/μL; hemoglobin (Hb), 13.5 g/dL; platelet, 9.8 × 10^4^/μL. Renal function was almost within normal range, but liver dysfunction was observed: total bilirubin, 2.8 mg/dL; asparate aminotransferase, 67 U/L; alanine transaminase, 35 U/L; alkaline phosphatase 332 U/L; γ-glutamyltranspeptidase, 190 U/L; lactate dehydrogenase (LDH) 415 U/L. Moreover, brain natriuretic peptide, a marker for heart failure, was markedly elevated to 1030.2 pg/mL. His chest computed tomography revealed pneumonia and heart failure.

**Table 1:** Laboratory data on admission in this subject.

**Variable**	**Result**	**Reference range**	**Variable**	**Result**	**Reference range**
**Peripheral blood**			**Blood biochemistry**		
White blood cells (/μL)	9,030	3,500–8,600	Total protein (g/dL)	6.4	6.6–8.1
Blast (%)	0.0	0.0–0.0	Albumin (g/dL)	3.1	4.1–5.1
Promyelo (%)	0.0	0.0–0.0	Globulin (g/dL)	3.3	2.2–3.4
Myelo (%)	0.0	0.0–0.0	Cholinesterase (U/L)	116	240–486
Meta (%)	0.0	0.0–0.0	Plasma glucose (mg/dL)	145	73–109
Band (%)	4.0	2.0–10.0	T. cholesterol (mg/dL)	114	142–248
Seg (%)	86.0	50.0–70.0	T. bilirubin (mg/dL)	2.8	0.4–1.5
Eosino (%)	0.0	1.0–5.0	AST (U/L)	67	13–30
Baso (%)	1.0	0.0–1.0	ALT (U/L)	35	10–42
Mono (%)	3.0	1.0–6.0	γ-GTP (U/L)	109	13–64
Lymph (%)	6.0	20.0–40.0	LDH (U/L)	415	124–222
Red blood cells (× 10^4^/μL)	441	435–555	ALP (U/L)	332	106–322
Hemoglobin (g/dL)	13.5	13.7–16.8	Creatinine (mg/dL)	0.91	0.65–1.07
Platelets (× 10^4^/μL)	9.8	15.8–34.8	BUN (mg/dL)	26	8–20
**Urinary test**			Uric acid (mg/dL)	4.5	3.7–7.8
Urinary pH	6.0	5.0–7.5	Sodium (mEq/L)	135	138–145
Urinary protein	2+	-	Potassium (mEq/L)	4.5	3.6–4.8
Urinary sugar	-	-	Chloride (mEq/L)	102	101–108
Urinary ketone body	-	-	Calcium (mg/dL)	7.9	8.8–10.1
Urinary blood	-	-	Amylase (U/L)	182	42–118
			CRP (mg/dL)	10.13	<0.14
			Procalcitonin (ng/mL)	0.55	0.00–0.05
			BNP (pg/mL)	1030.2	0.0–18.4

*T. cholesterol, total cholesterol; T. bilirubin, total bilirubin; AST, aspartate aminotransferase; ALT, alanine aminotransferase; γ-GTP, γ-glutamyltranspeptidase; LDH, lactate dehydrogenase; ALP, alkaline phosphatase; BUN, blood urea nitrogen; CRP, C-reactive protein; BNP, brain natriuretic peptide*.

After admission, we started antibiotics therapy for pneumonia (6.0 g/day of sulbactam/ampicillin), although pathogenic bacteria were not detected. In addition, we started to administer furosemide for acute heart failure. Next day, he had symptoms of jaundice and his total bilirubin level was elevated to 6.0 mg/dL. Five days after admission, his inflammation markers were further elevated: WBC, 22,450/μL; CRP, 11.15 mg/dL; procalcitonin, 4.57 ng/mL. Moreover, he had hemolytic anemia (RBC, 282 × 10^4^/μL; Hb, 9.0 g/dL; reticulocyte counts, 4.7 %; total bilirubin, 14.4 mg/dL; LDH, 1,328U/L; urinary blood, 3+; urinary bilirubin, 2+; urinary urobilinogen, 2+) and we examined the possible cause of hemolytic anemia. Hemolytic anemia-associated data were as follows: DAT, positive (3+); DAT (anti-IgG), (3+); DAT (anti-C3), (4+); indirect antiglobulin test, positive; cold agglutination, negative; haptoglobin, <10 mg/dL; cryoglobulin, negative. Another autoantibody including systemic lupus erythematosus were not detected. Urinary antigen of *streptococcus pneumoniae* and *legionella* were negative. Parvovirus B19, cytomegalovirus, both hepatitis B and C, both influenza A and B and *mycoplasma pneumonia* antigen and/or antibody were negative. Based on such findings including elevated procalcitonin level, we finally diagnosed him as severe warm-type AIHA associated with pneumoniae infection. We discussed the necessity of steroid therapy with hematologists, but finally we selected antibiotics therapy without steroid therapy because his AIHA was likely caused by infection. Since we failed to exclude the possibility of drug-induced AIHA, we stopped sulbactam/ampicillin and changed antibiotics therapy for pneumonia (1.0 g/day of meropenem and next day added 500 mg/day of levofloxacin). Seven days after admission, his severe leucocytosis was further aggravated: WBC, 75,230 /μL; CRP, 12.30 mg/dL; procalcitonin, 6.16 ng/mL. In addition, his hemolytic anemia was aggravated (RBC, 188 × 10^4^/μL; Hb, 6.4 g/dL; total bilirubin, 16.8 mg/dL; LDH, 3,236 U/L; urinary blood, 3+; urinary bilirubin, 2+; urinary urobilinogen, 3+). After then, he suddenly had cardiac arrest and respiratory failure, and finally died of multiple organ failure. Unfortunately, we failed to rescue him from severe AIHA.

Since the conditions in this subject with AIHA were exacerbated very rapidly, we examined the time course of various kinds of cytokines using residual serum after his death in order to clarify the possible mechanism for pneumonia-induced AIHA. As shown in Table 2, IL-18 level was very high before the onset of AIHA and further elevated within several days which was not accompanied by elevation IL-12 level, suggesting that this subject was under Th2-dominat conditions. IL-10 level was also elevated before the onset of AIHA without elevation IL-12 level. As the results, IL-10/IL-12 imbalance was observed, suggesting that this subject was under Th2-dominat conditions. Levels of TNF-α, IL-6 and IL-8 were also elevated before the onset of AIHA, whereas levels of INF-γ and IL-1β were within normal range.

**Table 2:** Inflammation markers, hemolytic markers and cytokine profiles on day 2, day 5, and day 7 after admission.

**Inflammation markers**	**Day 2 after admission**	**Day 5 after admission**	**Day 7 after admission**	**Reference range**
WBC (/μL)	10,910	22,450	75,230	3,500–8,600
Neut. (%)	87.6	76.3	60.6	<0.14
CRP (mg/dL)	11.62	11.15	12.3	0.00–0.05
PCT (ng/mL)	0.55	4.57	6.16	
**Hemolytic markers**	**Day 2 after admission**	**Day 5 after admission**	**Day 7 after admission**	**Reference Range**
RBC (× 10^4^/μL)	425	282	188	435–555
Hb (g/dL)	13.6	9.0	6.4	13.7–16.8
T. Bil (mg/dL)	6.0	14.4	16.8	0.4–1.5
ID.Bil (%)	-	38	32	48–70
LDH (U/L)	435	1,328	3,236	124–222
Urinary blood	-	3+	3+	-
Urinary bilirubin	-	2+	2+	-
Urinary urobilinogen	-	2+	3+	-
**Cytokines**	**Day 2 after admission**	**Day 5 after admission**	**Day 7 after admission**	**Reference Range**
IFN-γ (IU/mL)	0.3	<0.1	<0.1	<0.1
TNF-α (pg/mL)	8.99	8.73	13.2	0.6–2.8
IL-1β (pg/mL)	<10	<10	<10	<10
IL-6 (pg/mL)	143	44	1,210	<4.0
IL-8 (pg/mL)	55.2	47.8	151.0	<2.0
IL-10 (pg/mL)	37	8	25	<5
IL-12 (pg/mL)	<7.8	<7.8	<7.8	<7.8
IL-18 (pg/mL)	1,060	2,230	>5,000	126 ± 44.5

*WBC, white blood cell; Neut., neutrophil; CRP, C-reactive protein; PCT, procalcitonin; RBC, red blood cell; Hb, hemoglobin; T. Bil, total bilirubin; ID. Bil, indirect bilirubin; LDH, lactate dehydrogenase; IFN-γ, Interferon-γ; TNF-α, tumor necrosis factor-α; IL, interleukin; N/A, not applicable*.

## Discussion

AIHA is an autoimmune disease and is categorized as either primary or secondary AIHA. Secondary AIHA can result from many other illnesses and is sometimes caused by infection, especially by mycoplasma, viral pneumonia, and other respiratory infections. In addition, it is known that antibiotics can also induce AIHA. For example, penicillin-induced AIHA, which is mainly known as hapten type AIHA, can be induced by high-dose intravenous penicillin (9). In general, the onset of hapten type AIHA is subacute, although penicillin-type AIHA is categorized into not only hapten type AIHA but also mixed immune complex type and autoimmune type complicated with hapten type AIHA. Our patient mainly suffered from pneumonia infection on admission, because he did not have hemolytic anemia at that time. After admission his infectious markers were markedly elevated. As pneumonia was aggravated, he had an onset of warm-type AIHA which was exacerbated very rapidly. The diagnosis of AIHA was difficult. However, his DAT was positive for both anti-IgG and anti-C3 and indirect antiglobulin test was also positive. On the other hand, his cold agglutination was negative. Moreover, while it is known the onset of drug-induced AIHA is subacute, hemolytic anemia in this subject was immediately induced after starting sulbactam/ampicillin antibiotics therapy. Therefore, we thought that our patient suffered from warm-type AIHA, at least in part, induced by pneumonia.

Our patient did not get glucocorticoid therapy, which is the first-line treatment for AIHA. At first, we started treatment for pneumonia in this patient. However, we diagnosed him as AIHA 5 days after admission. We thought it was necessary to treat him with glucocorticoid therapy and discussed the necessity of glucocorticoid therapy with hematologists. However, hematologists did not agree to the usage of glucocorticoid, because if he suffered from viral infection, and his conditions became more severe and miserable. In addition, hematologists insisted on the importance of continuation of antibiotics therapy for infection disease, because they thought that his AIHA was likely caused by infection. Nonetheless, we still think glucocorticoid therapy is one of the important strategies for AIHA even if it is caused by infection disease, although we finally selected antibiotics therapy without glucocorticoid therapy in this case.

The precise mechanism for pneumonia-induced AIHA in this subject remains unknown. Since our patient's conditions with AIHA were very severe and exacerbated very rapidly, we investigated his cytokine profiles using residual serum after his death. IL-18 is known to function as Th1 cytokine together with IL-12 but function as Th2 cytokine without IL-12 (6). IL-18 level was high and further elevated within several days without elevation of IL-12 levels in this subject. These finding suggest that this subject was under Th2-dominant conditions which we assume led to rapid exacerbation of AIHA. Moreover, while IL-10/IL-12 imbalance is known to be an indicator of Th2-dominat conditions and a progressive marker of AIHA (7, 8), IL-10 levels were markedly elevated without elevation of IL-12 levels in this subject. This IL-10/ IL-12 imbalance also suggest that this subject was under Th2-dominant conditions and that AIHA was pretty progressive.

In addition, since it is known that mycoplasma pneumoniae increases production of IL-18, which leads to hemolytic anemia (9, 10), we assume that elevated IL-18 levels were associated with sever hemolytic anemia in this subject as well, although *mycoplasma pneumonia* antigen itself was not detected in this subject. It is also known that increased IL-10 levels lead to decrease in IFN-γ production and differentiation to Th2-dominant conditions in AHIA patients. Therefore, we assume that increased production of Th2 cytokines leads to augment the production of autoantibodies in subjects with AIHA induced by infection such as pneumonia. Taken together, it is likely that marked cytokine storm facilitated the aggravation of AIHA in this subject which rapidly worsened the conditions and finally led this subject to his death. This case report suggest that it is very important to promptly start steroid therapy for hemolytic anemia in subjects with infection-induced AIHA especially together with cytokine storm.

There are several strengths in this case report. First, pneumonia-induced AIHA is relatively rare and is not commonly observed in clinical practice. Second, there is few reports showing the alteration of cytokine profiles in pneumonia-induced AIHA. Third, there has been no report so far showing IL-10/IL-12 imbalance in infection-induced AIHA. Fourth, there has been no report showing that IL-18 levels are markedly elevated in subjects with AIHA. There are also several limitations in this report. First, although we think the diagnosis of AIHA was correct, it is difficult to precisely figure out the main cause of AIHA in this subject. We thought that our patient suffered from warm-type AIHA, at least in part, induced by pneumonia, but we think there was possibility of drug-induced AIHA and/or mixed conditions of various factors. Second, we did not treat our patient with glucocorticoid therapy due to above-described reasons. Third, since infection-induced AIHA was very rapidly exacerbated presumably due to marked cytokine storm in this subject and there was not enough time to make a closer examination, the precise mechanism for pneumonia-induced AIHA in this subject remains unknown. Fourth, since we examined his cytokine profiles using residual serum after his death, there might have been some error in measurement of cytokine levels. Finally, we failed to provide the information about pre-infection data of hemolytic anemia, because this patient visited our institution for the first time at that time.

Taken together, we should bear in mind that infection-induced AIHA can bring out hypercytokinemia, and that once marked cytokine storm is induced, AIHA is exacerbated very rapidly.

## Data Availability Statement

All datasets presented in this study are included in the article/supplementary material.

## Ethics Statement

Written informed consent was obtained from the individual(s) for the publication of any potentially identifiable images or data included in this article.

## Author Contributions

YI and TA researched data and wrote the manuscript. KK and RS researched data and contributed to the discussion. HK and KT reviewed the manuscript.

## Conflict of Interest

The authors declare that the research was conducted in the absence of any commercial or financial relationships that could be construed as a potential conflict of interest.
